# Kinematic and mechanical assessment of seated lumbar rotation manipulation: force, velocity and orientation in three dimensions

**DOI:** 10.3389/fbioe.2025.1651760

**Published:** 2025-09-30

**Authors:** Changxiao Han, Jiali Chen, Jinghua Gao, Congcong Wen, Haibao Wen, Xunlu Yin, Bochen Peng, Guangwei Liu, Liguo Zhu, Minshan Feng

**Affiliations:** ^1^ Wangjing Hospital, China Academy of Chinese Medical Sciences, Beijing, China; ^2^ Beijing University of Chinese Medicine, Beijing, China; ^3^ Beijing Key Laboratory of Digital Intelligence Traditional Chinese Medicine for Preventing and Treating Degenerative Bone and Joint Diseases, Beijing, China

**Keywords:** seated lumbar rotation manipulation, manual therapy, kinematics, mechanics, motion capture, somatotype

## Abstract

**Background:**

Seated lumbar rotation manipulation is widely used for low back pain, but lacks detailed biomechanical analysis. Understanding its biomechanical characteristics is crucial for therapists to improve comprehension and support education and research. The purpose of this study was to analyze the kinematic and mechanical parameters of Seated lumbar rotation manipulation.

**Methods:**

Sixty healthy volunteers underwent manipulation by experienced therapists. Three-dimensional movements, thrust velocity, and acceleration were measured using motion capture technology. Force parameters were recorded using pressure sensing gloves mounted on the therapist’s hands. Subgroup comparisons were conducted based on body mass index, and linear regression was used to analyse the relationship between force parameters and BMI (Body Mass Index). Finally, Pearson’s correlation test was employed to examine the correlation between the forces exerted by both hands during each procedure.

**Results:**

Kinematic analysis indicated that the angles in three directions were greatest for rotation, followed by lateral bending and flexion. Similarly, rotation was the dominant angular velocities, greater than lateral flexion and anteflexion. Furthermore, Preload duration (2.72 ± 0.10 s) and thrust duration (0.48 ± 0.04 s) were recorded. In terms of force, four key force metrics were calculated: preload force (58.99 ± 9.76 N), valley force (23.25 ± 6.24 N), thrust force (50.54 ± 9.63 N), and peak force (73.77 ± 11.06 N). While the preload rate (21.73 ± 4.66 N/s), thrust rate (106.30 ± 11.72 N/s), and the maximum torque (51.86 ± 7.52 N m) were determined. Subgroup analysis showed significant differences in force parameters by body types (P < 0.01). Linear regression revealed a positive correlation between BMI and force parameters (P < 0.05), and Pearson analysis indicated a significant correlation between forces exerted by both hands (P < 0.05).

**Conclusion:**

Seated lumbar rotation manipulation is characterized by long-lever, three-dimensional coupled movements with high-velocity, low-amplitude thrusts. Additionally, the force parameters are positively influenced by somatotype, and bilateral hand force exerts a synergistic effect. This valuable biomechanical quantification help comprehending the technique and supporting its educational and experimental settings.

## 1 Introduction

Manual therapy is popular worldwide and can effectively relieve pain caused by various musculoskeletal diseases (Bagagiolo et al., 2022; Asahi et al., 2020; Rodriguez-Merchan et al., 2020). Spinal manipulation, which usually involves high-velocity and low-amplitude thrusting, is the most widely used (Gevers-Montoro et al., 2021; Jenks et al., 2022). However, this process is complex and requires a high degree of coordination between the action and the amplitude, thus requiring operators to have ample experience, perception and psychological acumen. Spinal manipulation usually takes a long period of practice to master (Descarreaux and Dugas, 2010; Macanuel et al., 2005).

Some studies have documented instances of patient harm sustained during the course of spinal manipulation therapy (Heneghan et al., 2020; Rubinstein et al., 2019; Benyaich et al., 2019; Ryu et al., 2018; Swait and Finch, 2017; Gorrell et al., 2017), and factors such as lack of experience, blunt force output, excessive amplitude, and improper posture are the main causes of injury (Hansen et al., 2018; Bisiacchi and Huber, 2006; Lorme and Naqvi, 2003). The lack of standardized procedures decreases the efficacy of manipulations, results in bad patient experiences, and may lead to injury (Lamprecht and Padayachy, 2019). Therefore, quantifying the kinematic and force characteristics of spinal manipulation will help therapists understand the characteristics of manipulation, help in educational and experimental contexts and prevent injuries.

Spinal manipulations have some common components, including velocity, amplitude, leverage, direction, preload force, and thrust (Herzog, 2010; Triano et al., 2012). Abundant research has been conducted on the force and kinematic characteristics of spinal manipulations performed by manipulative therapists (Russell et al., 2022; Gorrell et al., 2022; du Rose et al., 2021; Derian et al., 2020; Engell et al., 2019; Corso et al., 2019; Arguisuelas et al., 2019). Motion capture technology, pressure sensors, pressure plates, and other devices have been used to measure the force transmission and changes in spinal position during spinal manipulation (Russell et al., 2022; Weiner et al., 2022; Anderst et al., 2018). Furthermore, there are differences in kinematics and kinetics among different spinal manipulations, body positions, and even body sizes. Therefore, for different spinal manipulations, based on the common biomechanical components of manipulations, further research on kinematic and kinetic parameters has important clinical significance.

Seated lumbar rotation manipulation is a popular traditional treatment for non-specific low back pain in China, as clinical trials and basic research have demonstrated its efficacy (Xie et al., 2021; Li et al., 2017). During the procedure, the subject is seated and the operator manipulates the spine with both hands to perform a long-lever movement in different directions of rotation. Additionally, seated lumbar rotation manipulation is within the scope of spinal manipulation and involves high-velocity and low-amplitude movements. However, the kinematic and force characteristics of seated lumbar rotation manipulation have not been quantified and analysed.

In addition to the basic biomechanical components mentioned above, this study specifically focused on somatotype variations. Understanding the relationship between somatotype and biomechanical parameters in seated lumbar rotation manipulation is crucial for several clinical and educational considerations. First, quantification of these parameters across different somatotypes could enable therapists to make evidence-based adjustments to manipulation force and velocity according to individual body types, potentially enhancing both treatment efficacy and safety. Second, such biomechanical data could serve as a foundation for developing standardized, somatotype-specific manipulation protocols, addressing the current lack of objective guidelines in clinical practice. Third, this knowledge is fundamental for educational settings, particularly in helping novice practitioners develop appropriate tactile sensitivity and force modulation skills for patients of varying body types. Therefore, this study aimed to investigate the thrust, velocity, and spatial orientation parameters associated with seated lumbar rotation manipulation using motion capture technology and pressure sensors, as well as to examine the impact of somatotype on these parameters. We hope the obtained information and data can guide therapists to better understand the characteristics of seated lumbar rotation manipulation and contribute to related research.

## 2 Methods

The primary objective of this study is to examine the kinematics and mechanics of manual quantification, with particular attention given to the consideration of safety and ethical issues. To this end, a selection of healthy volunteers was recruited for participation. Sixty healthy volunteers participated in this experiment; the volunteers had no lumbar spine disease and no contraindications to manipulation before enrolling in the study. The subjects, including 36 males and 24 females, ranged in age from 21–28 years. To determine the effect of somatotype on manipulation, the subjects were stratified by Chinese adult BMI classification standards with body mass index (BMI) (Zhou and Cooperative Meta-Analysis Group of the Working Group on Obesity in China, 2002), with 20 individuals in the ectomorph group (BMI<24), 20 individuals in the mesomorph group (BMI 24–28), and 20 individuals in the endomorph group (BMI 28). This study was approved by the Ethics Committee of Chinese Academy of Chinese Medical Sciences Wangjing Hospital (WJEC-KT-2021-055-P002).

### 2.1 Equipment

The motion capture system comprised 15 digital motion capture lenses (OptiTrack; Prime13 cameras; 1.3 million pixels; and 0.01-mm precision). OptiTrack is a high-precision motion capture system designed to capture three-dimensional movements of objects or humans (Yu et al., 2024; Ameler et al., 2019). Utilizing multiple cameras and reflective markers, it records spatial positions and motion trajectories in real-time. With its high sampling rate and precise spatial localization, OptiTrack is widely applied in motion analysis and sports science. The calibration process for the motion capture system was performed in accordance with the methodologies outlined in the two referenced studies (Feng et al., 2019; Liguo et al., 2017). The data acquisition software used was MOTIVEBODY, which is integrated with and provided as part of the OptiTrack system. Data were analyzed using Visual3D software (C-motion, USA). Visual3D is a software platform widely used for biomechanical research, motion analysis, and clinical evaluation (Ameler et al., 2019; Sinsurin et al., 2020; Lewis and Garibay, 2015). It processes three-dimensional data collected by motion capture systems like OptiTrack and offers functionalities for post-processing and analysis, including motion trajectory analysis, joint angle calculations, and kinematic and kinetic modeling. Visual3D enables detailed analyses of motion parameters, such as position, velocity, acceleration, and joint range of motion. The present study utilized a glove (Utility Model Patent No. 201620427405.8), self-developed by the investigators specifically for measurement of forces during massage and manipulation. This glove facilitates the mathematical extraction and analysis of the force characteristics of the manipulation process through multiple built-in pressure sensors, which have been employed in previous studies (Gao et al., 2018). The calibration process, which is integral to the glove’s functionality, involves the application of a known standard force to the pressure sensing system of the glove via a dedicated calibrator. This process enables the glove to measure forces with a high degree of accuracy. The standard force source generates force values of varying intensity, which are then applied to the glove’s pressure sensor. Subsequent to each application of the standard force, a comparison is made between the input standard force and the feedback data from the sensor. The response of the sensor is then adjusted according to the results of this comparison, ensuring that its output is consistent with the standard force value. The calibration process ensures that the glove is highly accurate under different operating conditions by applying the standard force several times and adjusting the pressure sensor. The hardware consists of mechanical sensors, biomechanical gloves, a synchronization signal circuit, a Bluetooth module, and a biomechanical data processing circuit. The software platform was developed in the VC2010 environment, with functions for device initialization, signal synchronization, data acquisition, and recording, as well as a reserved module for real-time data display. The system has a measurement precision of 0.1 N and a sampling frequency of 0–100 Hz. The motion capture system operated at a sampling frequency of 120 Hz, while the force measurement glove system recorded data at 50 Hz.

### 2.2 Manipulation

**FIGURE 1:**
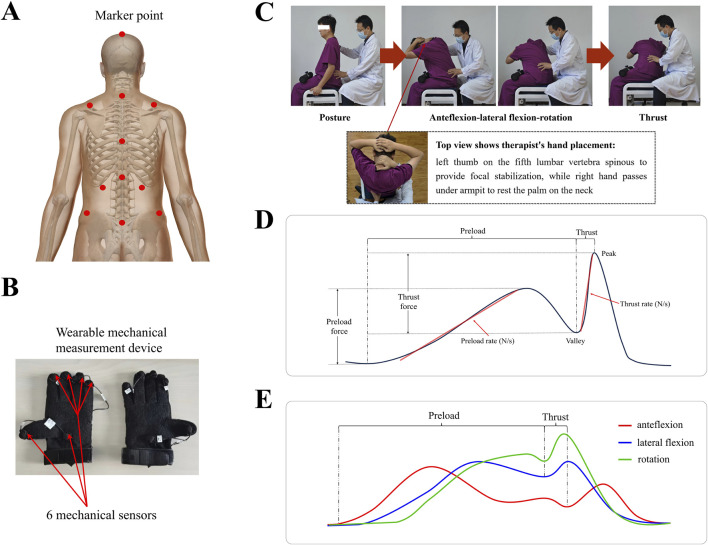
**(A)** Calibration of marker points for the motion capture system to capture three-dimensional movement directions. **(B)** Wearable force sensors placed at six points on the thumb, palm, and metacarpophalangeal regions to capture force data with a measurement accuracy of 0.1 N and a frequency range of 0–100 Hz. **(C)** The procedure of seated lumbar rotation manipulation, using the right side as an example, divided into two core phases: preload and thrust. **(D)** A schematic representation of typical force changes during the procedure with force measured at hand on the subject’s neck. **(E)** A schematic representation of typical three-dimensional angle changes during the procedure.

Taking the right-side seated lumbar rotation manipulation as an example, the subject sits upright with both legs fixed using a belt, and their hands placed behind the head. The therapist sits behind the subject, placing their left thumb on the spinous process at the spinous process of the fifth lumbar vertebra to provide focal stabilization, and passing their right hand under the subject’s right armpit to rest the palm on the subject’s neck. The subject’s upper body is manoeuvred to the right when performing the right-side seated lumbar rotation manipulation, and to the left when performing a left-side seated lumbar rotation manipulation. The procedure is divided into two phases: preload and thrust. During preload, the therapist guides the subject to flex forward while applying maximum lateral bending and rightward rotation. At the end of the preload phase, there is usually a brief pause with partial unloading of the force. The minimum force exerted during this process is referred to as the ‘valley force’. During the thrust phase, the therapist rapidly applies a rotational force with the right hand while simultaneously applying a pushing force on the spinous process with the left hand. A palpable movement of the spinous process is often felt, accompanied by an audible “click” (Figure 1C).

### 2.3 Procedures

This study was conducted at Chinese Academy of Chinese Medical Sciences Wangjing Hospital-Key Laboratory of Beijing of TCM Bone Setting. Before the experiment began, the instrument and the site were calibrated. The subjects wore specific clothes with 11 marked points for motion capture technology to record manual kinematic data. Specialized gloves mentioned above were utilized to record mechanical manipulation data, which could capture the interaction forces between the therapist’s hands and the contact points on the subject. After calibrating the instrument and site, the subject sat on a stool, and the manipulative therapist performed seated lumbar rotation manipulation. In the application of seated lumbar rotation manipulation, stabilization is specifically targeted at the spinous process of the fifth lumbar vertebra to ensure precise localization and effective force transmission. Each subject underwent both left- and right-sided manipulations, with the sequence of application determined by the parity of computer-generated random numbers to ensure randomization. All manipulations were performed by three experienced manipulative therapists (each with >10 years of experience). To ensure standardization, all therapists underwent a training session and demonstration protocol prior to data collection, and manipulation techniques were verified by the research team leader (Figure 1).

### 2.4 Data analysis

Using human anatomy and marker points, the changes in the three-dimensional vectors of the body during manipulation were analysed. Data were analysed using SPSS and MATLAB software. Quantitative data are expressed as mean ± standard deviation (M±SD). Paired sample t-tests were conducted to compare the manipulations between the left and right sides, while one-way analysis of variance (ANOVA) followed by the least significant difference (LSD) *post hoc* test was employed to assess differences across body types. Linear regression was used to analyse the correlation between BMI and manipulation parameters. *P* < 0.05 was considered statistically significant. The correlation of force data between the lumbar and cervical spine was analysed using the signal cross-correlation function.

Two independent coordinate systems were established within the motion capture system. Coordinate system 1 was defined by the C7 spinous process and the bilateral acromion points, while coordinate system 2 was determined by the bilateral posterior superior iliac spines and the L5 spinous process. The three-dimensional motion associated with the therapeutic procedure was recorded using the OptiTrack system. Following data extraction, Visual3D was utilized to calculate the movement angles and velocities in three-dimensional space. Displacement of markers on the therapist’s hands was used to determine the duration, velocity, and acceleration of the thrust. Data were collected using the custom measurement platform and exported to Excel for processing. Kinetic data were time-aligned with the kinematic data to identify key time points during the procedure. The preload force was defined as the highest force recorded during the positioning phase, typically at the end of the preload. The valley force was the lowest force recorded during the pause between the preload and thrust phases. Peak force was the maximum force recorded during the thrust, and the thrust force was calculated by subtracting the valley force from the peak force. The maximum torque was calculated based on the peak force exerted by the hand on subjects’ neck and the lever arm distance from the cervical contact point to the lumbar fulcrum during the thrust phase.

## 3 Results

A total of 60 participants successfully completed both the left- and right-side manipulations. No statistically significant differences were found in the comparison of kinematic and mechanical parameters between the left- and right-side manipulations (Supplementary Table S1). For each subject, the measurements from the two sides were averaged to obtain representative values. The force parameters (preload force, valley force, thrust force, peak force, preload rate, thrust rate, and maximum torque) were measured at the hand on the subject’s neck rather than on waist. The following sections present the detailed kinematic and mechanical characteristics of the seated lumbar rotation manipulation.

### 3.1 Kinematic parameters

**FIGURE 2:**
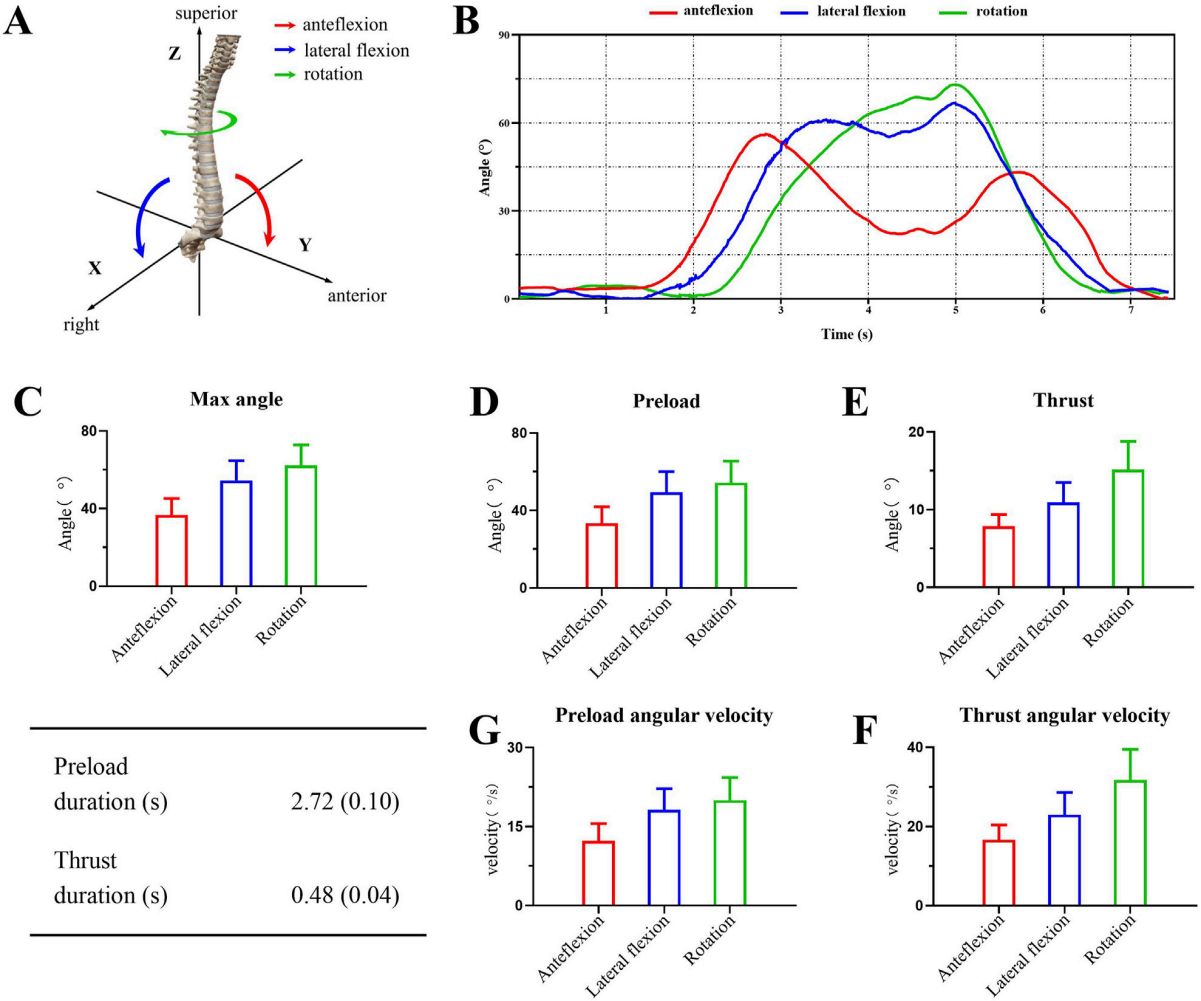
Kinematic description of seated lumbar rotation manipulation. **(A)** Definition of the three-dimensional coordinate system during the procedure. **(B)** Changes in three-dimensional directional angles for a single subject during manipulation. **(C)** The maximum angle changes in three-dimensional direction during the whole manipulation process. **(D, E)** Comparisons of angle changes in three-dimensional direction during different process. **(F, G)**. Comparisons of angular velocities in three-dimensional direction during different process.

Table 1 shows the kinematic parameters for seated lumbar rotation manipulation. During the preload phase, The angles of anteflexion, lateral flexion, and rotation were 33.41° ± 8.52°, 49.43° ± 10.68°, and 54.32° ± 11.16°, respectively. The thrust phase was mainly characterized by rotation (15.16° ± 3.63°), with minimal changes in anteflexion and lateral flexion (7.88° ± 1.48°, 10.93° ± 2.56°, respectively). During the manipulation process, the maximum angles of anteflexion, lateral flexion, and rotation were recorded as 36.75° ± 8.49°, 54.53° ± 10.18°, and 62.3° ± 10.54°, respectively. We selected the kinematic data from one participant to illustrate the typical three-dimensional movement pattern of seated lumbar rotation manipulation. Additionally, we determined the average time for the preload phase (2717.48 ± 100.07 ms), the average time for the thrust phase (478.8 ± 43.34 ms) and angular velocities in three dimensions (Figure 2). All kinematic parameters showed no statistically significant differences when compared across different body types (Supplementary Table S2).

**TABLE 1:** Kinematic parameters of seated lumbar rotation manipulation.

Direction	Angle (°)			Angular velocity (°/s)	
	Preload	Thrust	Maximum	Preload	Thrust
Anteflexion	33.41 (8.52)	7.88 (1.48)	36.75 (8.49)	12.32 (3.24)	16.65 (3.75)
Lateral flexion	49.43 (10.68)	10.93 (2.56)	54.53 (10.18)	18.21 (3.99)	23.01 (5.61)
Rotation	54.32 (11.16)	15.16 (3.63)	62.3 (10.54)	20.03 (4.29)	31.79 (7.72)

Data are presented as mean (standard) deviation. The table presents the angles (in degrees) and angular velocities (in degrees per second) for anteflexion, lateral flexion, and rotation during the preload, thrust, and maximum phases of the seated lumbar rotation manipulation.

### 3.2 Force parameters

**FIGURE 3:**
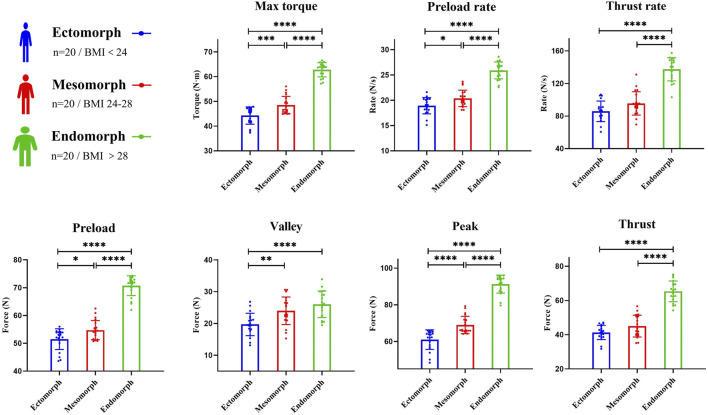
Comparison of force parameters (Max torque, preload rate, thrust rate, preload force, valley force, peak force, thrust force) between groups stratified by BMI. The force data presented are measured at the hand on the subject’s neck. Asterisks indicate statistically significant differences between groups (*P < 0.05; **P < 0.01; ***P < 0.001; ***P < 0.0001).

Table 2 shows the force parameters of seated lumbar rotation manipulation. Based on the characteristics of the technique, four key forces were selected, and the average values were determined: preload force (58.99 ± 9.16 N), valley force (23.25 ± 4.77 N), thrust force (50.54 ± 11.99 N), and peak force (73.77 ± 13.83 N). Combined with kinematic data, we calculated the preload force rate (21.73 ± 3.43 N/s), thrust force rate (106.30 ± 26.31 N/s), and maximum torque (51.86 ± 8.62 N m). Comparisons between different BMI groups revealed statistically significant differences in force, force rate, and torque among the groups (P < 0.001). Further pairwise comparisons indicated that the difference in valley force between the medium and high BMI groups was not significant (P = 0.251), nor was the difference in thrust force between the low and medium BMI groups (P = 0.098), or the difference in thrust force rate between the low and medium BMI groups (P = 0.073). All other parameters showed significant differences between groups (P < 0.001) (Figure 3).

**TABLE 2:** Force parameters of seated lumbar rotation manipulation.

Manipulation parameters	Overall mean	Stratification based on BMI			P value
		Ectomorph (n = 20)	Mesomorph (n = 20)	Endomorph (n = 20)	
		(BMI < 24)	(BMI 24–28)	(BMI > 28)	
preload force (N)	58.99 (9.16)	51.52 (3.73)	54.75 (3.45)	70.71 (3.56)	0.001
Valley force (N)	23.25 (4.77)	19.71 (3.52)	23.99 (4.34)	26.04 (4.18)	0.001
Thrust force (N)	50.54 (11.99)	41.29 (4.16)	45.03 (6.37)	65.30 (6.06)	0.001
Peak force (N)	73.77 (13.83)	61.00 (5.35)	69.02 (4.74)	91.30 (4.90)	0.001
Preload rate (N/s)	21.73 (3.43)	18.93 (1.61)	20.37 (1.63)	25.90 (1.67)	0.001
Thrust rate (N/s)	106.30 (26.31)	85.86 (12.59)	95.57 (14.32)	137.47 (14.22)	0.001
Max torque (N*m)	51.86 (8.62)	44.27 (3.47)	48.51 (3.50)	62.79 (2.87)	0.001

Data are presented as mean (standard) deviation. The force data presented are measured at the hand on the subject’s neck. The table shows the overall mean values and stratified data based on body mass index (BMI) for three groups: ectomorph (BMI < 24), mesomorph (BMI, 24–28), and endomorph (BMI > 28). Statistical significance (P-value) is indicated for comparisons between BMI, groups.

**FIGURE 4:**
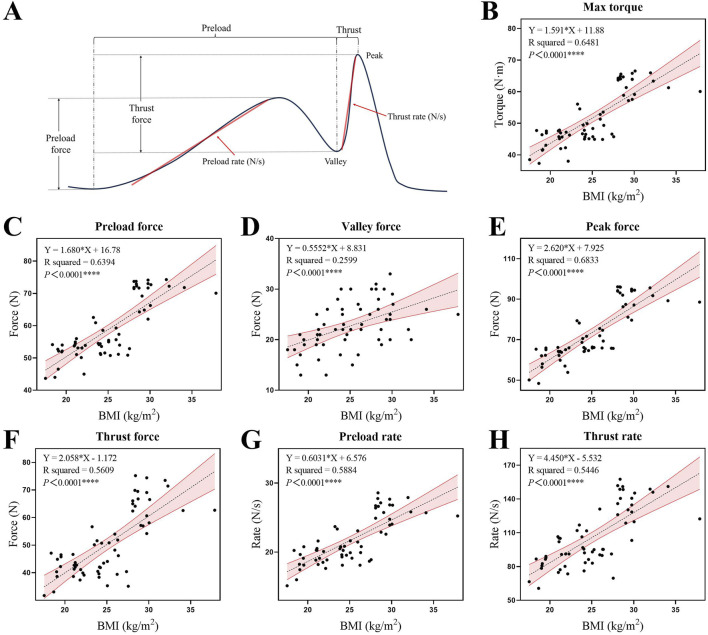
**(A)** Schematic representation of typical force changes during the procedure, with annotations for the identification of different force parameters. **(B–H)** Linear regression diagrams depicting the relationships between BMI and various force parameters: max torque, preload force, valley force, peak force, thrust force, preload rate, and thrust rate. The force data presented are measured at the hand on the subject’s neck. The shaded area represents the 95% confidence interval for the regression line.

**FIGURE 5:**
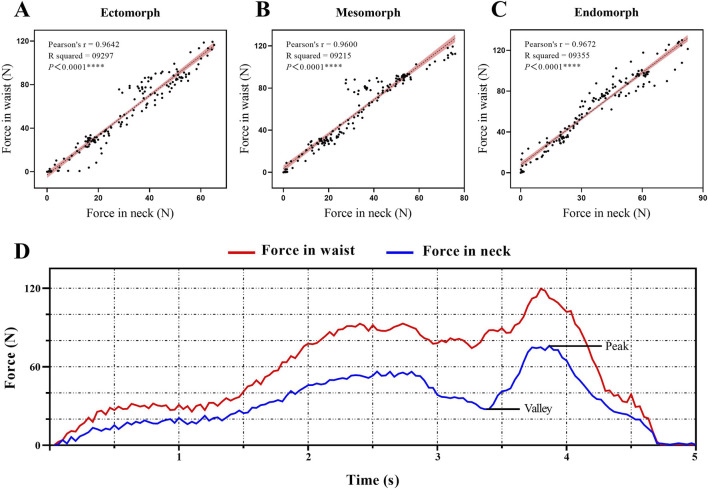
**(A–C)** Pearson correlation diagrams of bilateral hand force parameters for typical individuals in different BMI groups. The shaded area represents the 95% confidence interval for the regression line. **(D)** Changes in bilateral hand force parameters during a single manipulation procedure.

Linear regression analysis demonstrated a positive correlation between BMI and force parameters (P < 0.05). The strongest correlation was between BMI and peak force (*R* ^2^ = 0.6833, P < 0.0001), while the weakest correlation was between BMI and valley force (*R* ^2^ = 0.2599, P < 0.0001) (Figure 4). Based on the coordinated hand force characteristic of the technique, Pearson correlation analysis showed a significant positive correlation between the forces exerted by both hands (force in neck and force in waist) during the procedure (r = 0.990, P < 0.001). We also described the force variation curves for both hands during a single manipulation and the typical Pearson correlation of hand forces in different BMI groups (Figure 5). Time-domain analysis revealed high bilateral hand coordination during manipulation procedure, with force in neck peaking at 73.77 ± 13.83 N and force in waist at 119.14 ± 13.83 N, demonstrating a peak force time delay of 66.71 ± 49.02 ms.

## 4 Discussion

As a representative method for the treatment of low back pain, seated lumbar rotation manipulation has been clinically proven to be effective in relieving muscle spasms, releasing adhesions, correcting spinal and joint disorders, reducing local pain and restoring functional activities involving the lumbar spine (Xie et al., 2021; Li et al., 2017; Masharawi et al., 2020). Therapists must not only exert certain forces on the treated area but also master certain skills (Li et al., 2017). The analysis results suggest that the kinematic and force characteristics of seated lumbar rotation manipulation differ significantly from other rotation-based techniques. Compared to the supine lumbar rotation manipulation, which is widely employed in clinical practice (Du et al., 2016; Ghasabmahaleh et al., 2021; Gibbons and Tehan, 2001), the seated lumbar rotation manipulation leverages the majority of the subject’s upper body to apply rotational force. The point of action for guiding spinal movement is positioned at the cervical spine, while the lumbar segments serve as the true fulcrum of rotation. This biomechanical arrangement results in a longer and more effective lever arm, enhancing the mechanical efficiency of the technique. Concurrently, the relatively stable pelvis enables enhanced focalisation of force on the target segment, alongside greater flexibility in the direction and amplitude of rotation. The range of adjustment encompasses the entire lumbar spine, thus providing a comprehensive approach to treatment. Conversely, supine lumbar rotational manipulation employs force through the subject’s lower body, resulting in a shorter lever arm and greater stability, which is generally suitable for adjustments to the lower segments of the lumbar spine (Bell et al., 2017).

This technique emphasizes the application of smaller forces over a longer duration. Previous studies have indicated that other High Velocity Low Amplitude spinal manipulation techniques typically require thrust forces exceeding 100 N to achieve optimal outcomes (Colloca et al., 2003; Keller and Colloca, 2000; Krouwel et al., 2010). However, in this technique, the relatively low absolute force (50.54 ± 11.99 N) applied by the therapist during the thrust, likely facilitated by the use of a longer lever arm, still results in effective joint mobilization due to the extended duration of force application to the torso. These findings suggest that seated lumbar rotation manipulation is a long-lever technique, characterized by the combination of a long lever arm and smaller forces. This allows the therapist to achieve an effective maximum torque while maintaining greater control over the movement, thereby enhancing the precision of the manipulation. The relatively low absolute force exerted by the therapist may partly explain the comparatively low force rate observed in our study. Previous research on lumbar manipulation has indicated that force rates associated with effective therapeutic outcomes are typically higher than the values (106.30 ± 26.31 N/s) measured in this study (Reed et al., 2013). As a critical parameter in lumbar manipulation, a higher force rate is generally associated with enhanced neuromuscular activation (Pickar et al., 2007; Sung et al., 2005). However, it is important to emphasize that the force rate measured in this study reflects the force exerted by the therapist’s guiding hand at the cervical region. When amplified through the long lever arm, the actual force rate applied to the target lumbar segment may be substantially higher. Accordingly, on the basis of the force application characteristics under consideration, it is also proposed that the manipulation be categorised as a long lever High Velocity Low Amplitude. Given that the primary aim of this study was the preliminary quantification of kinematic and force data during seated rotational manipulation, a more detailed investigation of this aspect was beyond its scope. We anticipate addressing this issue in future research.

A previous study (Kos et al., 2019) found a significant correlation between the height of subjects with degenerative lumbar spondylolisthesis and the maximum velocity and maximum acceleration of seated lumbar rotation manipulation. Considering that seated lumbar rotation manipulation is a long-lever thrust-based manipulation, it involves substantial passive lumbar motion during the procedure. Therefore, body type (BMI) may be an important factor affecting the kinematics and mechanics of this manipulation. However, the results of this study showed that the kinematic parameters of manipulation were less affected by body size. In comparison to body size, we posit that degenerative spinal changes, such as osteophyte formation, which result in restricted flexion, lateral bending, and rotational motion of the spine, are more likely to influence the kinematic characteristics during seated rotational manipulation (LaPelusa and Bordoni, 2025; Ka et al., 2021; Jaumard et al., 2011; Rustenbu et al., 2020; Li et al., 2011). We anticipate further investigation into this aspect in future studies.

The mechanical results of this study indicate that there is a strong correlation between the force in neck and force in waist, indicating a very high degree of cooperation between the two hands during this manipulation. The time delay between the application of force by the two hands was less than 0.1 s, further indicating the necessity of a high degree of coordination between hands when performing seated lumbar rotation manipulation. Analysis of the actual procedure indicated that the force in neck and force in waist occurred almost simultaneously. Prior to the execution of the thrust, the subject’s trunk must be flexed and rotated to a relatively limiting and fixed position so that subsequentforces can be applied with greater precision to the target segment. This process requires mutual feedback and coordination through the therapists high sensory-motor functions in order to understand and adjust the required force and amplitude of the two-handed rotation of the subject. Furthermore, the results also showed that the peak force in neck was significantly smaller than the the peak force in waist. Seated lumbar rotation manipulation operates as a lever-based biomechanical system, where the target lumbar vertebra serves as the fulcrum. The therapist’s hands apply forces at two different points along this lever system. The force in neck, applied at a greater distance from the fulcrum, functions as the long-arm component of the lever and is primarily responsible for guiding the patient’s trunk through the rotational movement. Due to the mechanical advantage provided by the longer lever arm, this guiding force requires relatively low magnitude to achieve the desired motion. Conversely, the force in waist is applied directly at or near the fulcru, acting as the short-arm component that provides stabilization and resistance. According to lever mechanics, this stabilizing force must be of greater magnitude to counterbalance the rotational moment created by the cervical force. This biomechanical arrangement allows the manipulation to achieve effective joint mobilization through the coordinated application of a small guiding force over a long lever arm and a larger stabilizing force at the fulcrum.

There are limitations to this study. This experiment is an *in vivo* study, which focuses more on the kinematic parameters of the lumbar region as a whole and does not allow for a precise study of the characteristics of the movements that actually occur in the vertebral body. Another limitation is that compensatory movements of the thoracic spine inevitably occur due to its anatomical and biomechanical coupling with the lumbar spine. Since the present study aimed to provide an overall quantification of the manipulation kinematics, we did not separately measure thoracic and lumbar motions. In the future, it may be possible to conduct further correlation analysis of lumbar spine displacement using spine specimens. In this study, only healthy people were selected as subjects. Additionally, whether participant feedback or psychological expectations affected the kinematic or force data cannot be determined. Finally, this study only analyzed the effect of body size on manipulation parameters, and therefore, further detailed analysis of the characteristics of therapists and subjects should be performed.

## 5 Conclusion

The main findings of this study indicate that seated lumbar rotation manipulation is a compound three-dimensional movement involving anteflexion, lateral flexion, and rotation, characterized by high-velocity, low-amplitude movements and an extended lever arm, which significantly enhances its mechanical efficiency. In addition, the synergistic force exerted by both hands is mechanically characteristic of seated lumbar rotation manipulation, and the peak thrust force increases with increasing BMI. The results of this study will help manipulative therapists better understand the procedural characteristics of seated lumbar rotation manipulation to help in educational and experimental contexts.

## Data Availability

The raw data supporting the conclusions of this article will be made available by the authors, without undue reservation.
