# Supplementary material for: Kinematic and mechanical assessment of seated lumbar rotation manipulation: force, velocity and orientation in three dimensions
Source: Front Bioeng Biotechnol. 2025 Sep 30;13:1651760. doi: 10.3389/fbioe.2025.1651760 (PMC12518278; doi:10.3389/fbioe.2025.1651760)
Supplement: Supplementary file 1 [file Table1.docx]

**Table S1.** Kinematic parameters of seated lumbar rotation manipulation: comparison between left- and right-sided manipulations

| parameters | left | right | average | P value |
| --- | --- | --- | --- | --- |
| Angle (°)- Preload |  |  |  |  |
| Anteflexion | 33.39 (8.67) | 33.43 (8.51) | 33.41（8.52） | 0.871 |
| Lateral flexion | 49.16 (10.42) | 49.70 (11.52) | 49.43（10.68） | 0.416 |
| Rotation | 54.66 (10.90) | 53.98 (11.95) | 54.32（11.16） | 0.294 |
| Angle (°)- Thrust |  |  |  |  |
| Anteflexion | 7.85 (1.48) | 7.91 (1.50) | 7.88（1.48） | 0.252 |
| Lateral flexion | 10.92 (2.61) | 10.94 (2.54) | 10.93（2.56） | 0.858 |
| Rotation | 15.06 (3.62) | 15.27 (3.73) | 15.16（3.63） | 0.611 |
| Angle (°)-Maximum |  |  |  |  |
| Anteflexion | 36.92 (8.40) | 36.58 (8.85) | 36.75（8.49） | 0.686 |
| Lateral flexion | 54.23 (10.19) | 54.83 (10.44) | 54.53（10.18） | 0.557 |
| Rotation | 62.77 (10.62) | 61.84 (11.05) | 62.3（10.54） | 0.373 |
| Velocity (°/s)- Preload |  |  |  |  |
| Anteflexion | 12.29 (3.21) | 12.35 (3.27) | 12.32（3.24） | 0.781 |
| Lateral flexion | 18.39 (4.03) | 18.03 (3.97) | 18.21（3.99） | 0.446 |
| Rotation | 20.16 (4.34) | 19.91 (4.26) | 20.03（4.29） | 0.629 |
| Velocity (°/s)- Thrust |  |  |  |  |
| Anteflexion | 16.51 (3.71) | 16.79 (3.81) | 16.65（3.75） | 0.507 |
| Lateral flexion | 22.86 (5.70) | 23.17 (5.54) | 23.01（5.61） | 0.579 |
| Rotation | 31.57 (7.63) | 32.01 (7.82) | 31.79（7.72） | 0.502 |
|  |  |  |  |  |
| preload force (N) | 58.95 (9.50) | 59.03 (9.23) | 58.99 (9.16) | 0.871 |
| Valley force (N) | 23.19 (4.67) | 23.31 (4.94) | 23.25 (4.77) | 0.416 |
| Thrust force (N) | 50.76 (11.75) | 50.32 (12.44) | 50.54 (11.99) | 0.294 |
| Peak force (N) | 73.26 (14.01) | 74.28 (14.48) | 73.77 (13.83) | 0.252 |
| Preload rate (N/s) | 21.72 (3.54) | 21.74 (3.40) | 21.73 (3.43) | 0.858 |
| Thrust rate (N/s) | 105.43 (27.77) | 107.17 (25.87) | 106.30 (26.31) | 0.206 |
| Max torque (N*m) | 52.07 (8.68) | 51.65 (8.87) | 51.86 (8.62) | 0.316 |

Data are presented as mean (standard) deviation. The “Average” column represents the combined results of left- and right-sided manipulations. Left and Right columns show the respective values for manipulations performed on each side. Paired sample t-tests were conducted to compare the left- and right-sided manipulations for each indicator. No statistically significant differences were found between sides (all P > 0.05).

**Table S2.** Kinematic parameters of seated lumbar rotation manipulation in different body size

| parameters | Ectomorph (n=20) (BMI 19-24) | Mesomorph (n=20) (BMI 24-28) | Endomorph (n=20) (BMI>28) | P value |
| --- | --- | --- | --- | --- |
| Angle (°)- Preload |  |  |  |  |
| Anteflexion | 31.72 (9.84) | 35.43 (7.65) | 33.08 (8.92) | 0.196 |
| Lateral flexion | 47.25 (12.15) | 51.86 (9.88) | 49.17 (11.34) | 0.243 |
| Rotation | 56.83 (12.47) | 51.94 (10.25) | 54.73 (11.68) | 0.158 |
| Angle (°)- Thrust |  |  |  |  |
| Anteflexion | 7.43 (1.72) | 8.29 (1.26) | 7.94 (1.58) | 0.113 |
| Lateral flexion | 10.15 (3.08) | 11.64 (2.15) | 10.98 (2.74) | 0.089 |
| Rotation | 14.27 (4.23) | 16.18 (3.12) | 15.05 (3.89) | 0.151 |
| Angle (°)-Maximum |  |  |  |  |
| Anteflexion | 35.68 (9.42) | 38.21 (7.83) | 36.75 (8.94) | 0.357 |
| Lateral flexion | 52.47 (11.78) | 56.92 (8.94) | 54.36 (10.62) | 0.224 |
| Rotation | 64.85 (12.35) | 59.47 (9.73) | 62.19 (11.28) | 0.172 |
| Velocity (°/s)- Preload |  |  |  |  |
| Anteflexion | 11.38 (3.86) | 13.21 (2.74) | 12.47 (3.52) | 0.138 |
| Lateral flexion | 17.52 (4.73) | 19.18 (3.42) | 18.09 (4.28) | 0.293 |
| Rotation | 19.14 (5.12) | 21.37 (3.68) | 19.86 (4.75) | 0.174 |
| Velocity (°/s)- Thrust |  |  |  |  |
| Anteflexion | 15.73 (4.25) | 17.84 (3.18) | 16.52 (4.06) | 0.126 |
| Lateral flexion | 21.68 (6.47) | 24.73 (4.83) | 22.95 (6.12) | 0.165 |
| Rotation | 29.94 (8.92) | 34.28 (6.74) | 31.61 (8.45) | 0.142 |

Data are presented as mean (standard) deviation. One-way ANOVA were conducted to compare the different body size group. No statistically significant differences were found between groups (all P > 0.05).
